# Accelerated Systemic Autoimmunity in the Absence of Somatic Hypermutation in 564Igi: A Mouse Model of Systemic Lupus with Knocked-In Heavy and Light Chain Genes

**DOI:** 10.3389/fimmu.2017.01094

**Published:** 2017-09-13

**Authors:** Gabrielle McDonald, Carlos O. Medina, Monika Pilichowska, John F. Kearney, Reiko Shinkura, Erik Selsing, Henry H. Wortis, Tasuku Honjo, Thereza Imanishi-Kari

**Affiliations:** ^1^Department of Integrative Physiology and Pathobiology, Sackler School of Graduate Biomedical Sciences, Tufts University School of Medicine, Boston, MA, United States; ^2^Department of Pathology and Laboratory Medicine, Tufts Medical Center, Boston, MA, United States; ^3^Department of Microbiology, University of Alabama, Birmingham, AL, United States; ^4^Department of Immunology, Nagahama Institute of Bioscience and Technology, Nagahama, Japan; ^5^Department of Immunology and Genomic Medicine, Graduate School of Medicine, Kyoto University, Kyoto, Japan

**Keywords:** activation-induced cytidine deaminase, somatic hypermutation, class switch recombination, systemic lupus erythematosus, autoantibodies, pathogenic antibodies, B cell central tolerance, RNA-specific antibodies

## Abstract

564Igi mice have knocked-in immunoglobulin (Ig) heavy (H) and light (L) chain genes that encode an autoantibody recognizing RNA. Previously, we showed that these mice produce pathogenic IgG autoantibodies when activation-induced deaminase (AID) is expressed in pre-B and immature B cells but not when it is expressed only in mature B cells. AID has two functions; it is necessary for somatic hypermutation (SHM) and class switch recombination (CSR). To determine the role of each of these functions in the generation of pathogenic autoantibodies, we generated 564Igi mice that carry a mutant AID-encoding gene, *Aicda* (*Aicda*^G23S^), which is capable of promoting CSR but not SHM. We found that 564Igi *Aicda*^G23S^ mice secreted class-switched antibodies (Abs) at levels approximately equal to 564Igi mice. However, compared to 564Igi mice, 564Igi *Aicda^**G23S**^* mice had increased pathogenic IgG Abs and severe systemic lupus erythematosus-like disease, including, glomerulonephritis, and early death. We suggest that in 564Igi mice SHM by AID changes Ig receptors away from self reactivity, thereby mitigating the production of autoantibody, providing a novel mechanism of tolerance.

## Introduction

Various systemic autoimmune diseases exhibit a high frequency of antibodies (Abs) that recognize nucleic acid antigens, suggesting that nucleic acids may have properties that promote the breaking of B-cell tolerance. Autoantibodies that bind DNA are more commonly studied, but RNA-binding Abs are frequent in systemic lupus erythematosus (SLE) patients and, unlike anti-DNA, correlate with disease severity (1). In order to understand the mechanisms involved in the production of pathogenic autoantibodies, we produced the 564Igi mouse model of SLE; this strain has knocked-in immunoglobulin (Ig) heavy (H) and light (L) chain genes that encode an autoantibody that recognizes RNA. 564Igi mice have only low-levels of serum anti-RNA IgM Abs, consistent with the fact that their splenic B cells are anergic and apparently tolerized (2). Unexpectedly, however, IgG2a/IgG2b anti-RNA Abs are found at high levels in the sera of 564Igi mice and IgG Abs that exhibit the 564 idiotypic marker (Id^+^) are found in the glomeruli, resulting in an SLE-like disease (2). The production of these anti-RNA Abs is toll-like receptor 7 (TLR7) and TLR8 dependent (2, 3). As these anti-RNA IgG Abs are pathogenic, they resemble those found in SLE patients having RNA-specific Abs, indicating that the 564Igi mouse is an excellent model for unraveling the developmental mechanisms that give rise to autoantibodies that recognize nucleic acids, thereby providing insight into potential new targets for intervention in disease progression.

The fact that B cell tolerance is breached in 564Igi mice (2) despite the absence of detectable, non-anergic IgM^+^Id^+^ B cells in the periphery, led to the search for the cells responsible for the production of pathogenic Id^+^ Abs in these mice. If anergic mature B cells *in vivo* are unable to differentiate into antibody-producing cells, then some Id^+^ B cells must be able to evade anergy to produce the pathogenic Id^+^ Abs. Thus, there must be a breach in central and/or peripheral tolerance.

The bone marrow (BM) is the site where autoreactive, surface IgM^+^ adult origin B cells first encounter and respond to self-antigen by upregulating expression of the recombination-activating gene (RAG) and initiating receptor editing (4–8). Activation-induced cytidine deaminase (*Aicda*/AID), which is required for somatic hypermutation (SHM) and class switch recombination (CSR) of Ig genes, is highly expressed in mature germinal center (GC) B cells, where it enables the generation of high affinity Abs to environmental antigens (9). Our lab and others (10–16) have discovered that, in mice and humans, expression of activation-induced deaminase (AID) can be induced in developing B cells resulting in both CSR and SHM. These findings are provocative because they suggest possible roles for AID in B cell tolerance and B cell-dependent autoimmunity.

The production of IgG autoantibodies in humans is crucial for the pathogenesis of SLE (17–20). We previously found that AID is necessary for the production of pathogenic antibody in 564Igi mice (3, 21). Surprisingly, AID activity in mature B cells is not sufficient for the production of autoreactive IgG (21). We found that *Aicda* expression and CSR in developing B cells are critical for the production of pathogenic IgG autoantibodies in these mice (3, 21). Of note, *Aicda* expression is elevated in pre-B and immature BM B cells from 564Igi mice (21).

A likely explanation for the requirement for AID activity in developing B cells to produce autoantibodies in 564Igi stems from examination of mechanisms of central B cell tolerance. During B cell development, before B cell maturity, strong cross-linking of IgM BCR can signal the induction of tolerance by deletion, anergy, or receptor editing mechanisms (22, 23). In contrast, IgG BCR cross-linking induces signaling events that lead to B-cell activation, proliferation, and differentiation (24, 25). Because the signal pathways downstream of surface IgM and IgG differ, CSR can alter the post-activation fate of a B cell. We suggest that this change in signaling means that CSR from IgM to IgG in developing B cells allows self-reactive B cells to evade tolerance mechanisms.

It has been shown that self-reactive mature B cells *in vitro* can be activated through dual BCR and endosomal TLR signaling (26, 27) and that dual ligation of these receptors can induce AID expression and CSR (28). It has also been shown that immature B cells in the BM can be activated by similar mechanisms (21, 29). Self-nucleic acid, which is abundant in the BM microenvironment (30), would have the potential to bind and stimulate the self-reactive BCRs found on the surface of many immature B cells (31). Once recognized, this self-antigen could be endocytosed and delivered to the endosome (32). In the endosome, TLR7 and TLR8, which recognize RNA, could potentially be stimulated by this internalized self-antigen (32), leading to the expression of *Aicda*, followed by premature CSR and the production of pathogenic IgG autoantibodies.

While AID-mediated CSR can facilitate the breaching of tolerance, several reports demonstrate that the absence of AID increases the production of self-reactive Abs, suggesting that AID also contributes to B cell tolerance (33, 34). One potential mechanism by which AID can mediate tolerance is through SHM of self-reactive Ig genes.

However, since a deficiency of AID results in the production of only IgM Abs, the role of AID in the prevention of pathogenic IgG antibody production remains unknown. In order to test this hypothesis, we developed a novel mouse 564Igi model with a knock-in mutation (G23S) in the *Aicda* gene (designated 564Igi *Aicda^G23S^*). This mutation leads to deficient SHM activity but has no apparent effect on CSR (35) and, thereby, separates the two AID functions. We used this mouse model to definitively determine the specific roles of SHM in central B cell tolerance.

## Results

### 564Igi *Aicda^G23S^* and 564Igi Mice Have Equivalent Numbers of Spleen and BM B Cells

**Figure 1:**
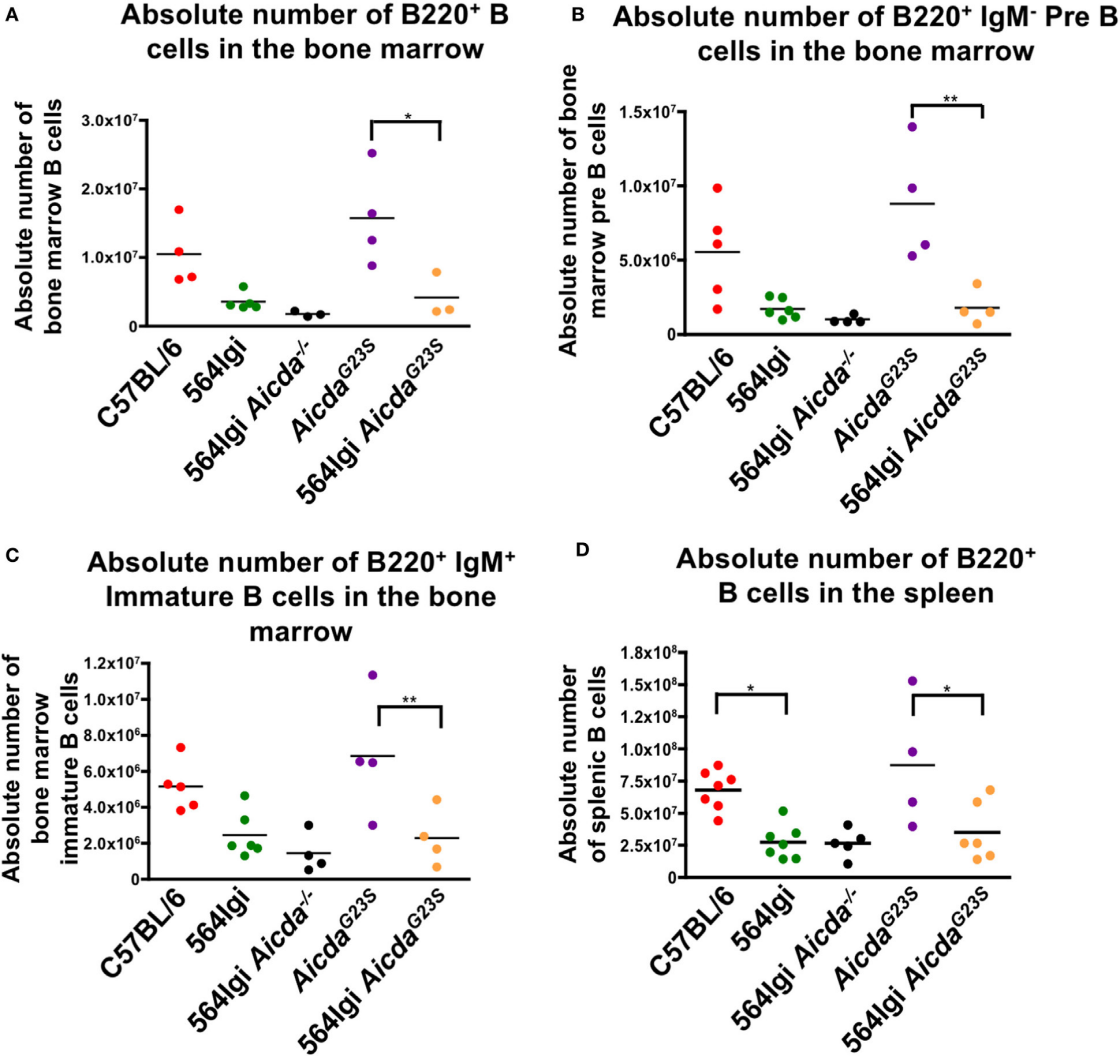
564Igi *Aicda^G23S^* and 564Igi mice have equal numbers of splenic and bone marrow (BM) B cells. Total BM **(A–C)** or spleen **(D)** cell suspensions from the indicated mice were stained with fluorescent antibodies and analyzed by flow cytometry to detect B220^+^ B cell **(A,D)** B220^+^ IgM^−^ pre B cells **(B)** and B220^+^ IgM^+^ immature B cells **(C)**. Shown is the absolute number of live cells in each animal with the mean shown as a horizontal line. Each point represents an individual animal.

In order to verify that a lack of SHM does not affect B cell development, we stained whole spleen and BM cell suspensions for B cells at various developmental stages. Here, we show that the introduction of anti-RNA antibody-coding Ig genes caused a decrease in both BM and splenic B cells in 564Igi mice (Figures 1A–D), confirming previously published results (21). Because 564Igi mice express the self-reactive 564 receptor, many B cells may be clonally deleted, accounting for the reduction in splenic and BM B cells. Similarly, 564Igi *Aicda*^G23S^ mice had significantly fewer BM and splenic B cells than *Aicda*^G23S^ controls (Figures 1A–D), likely due to the increase in clonal deletion caused by the presence of the 564 knock-in. However, there is no difference in the number of splenic or BM B cells found in 564Igi and 564Igi *Aicda*^G23S^ mice (Figures 1A–D). Therefore, the lack of SHM does not affect B cell development in 564Igi mice.

### Elevated Levels of *Aicda* Are Found in the B Cells of 564Igi and 564Igi *Aicda*^G23S^ Mice

**Figure 2:**
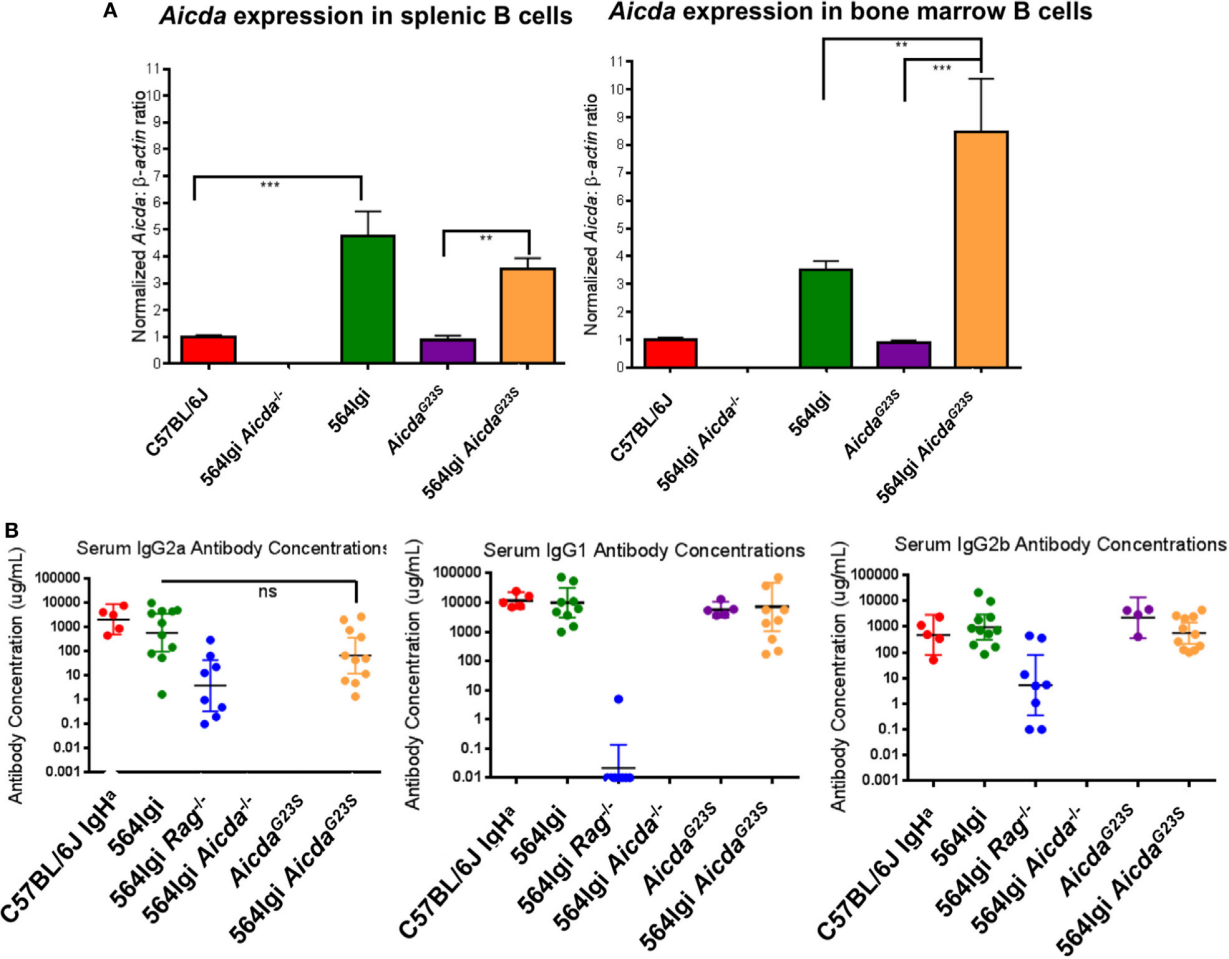
B cells from 564Igi *Aicda^G23S^* mice expressed more *Aicda* and similar levels of IgG antibodies than 564Igi mice. **(A)** Quantitative real-time PCR analysis of *Aicda* expression in B220^+^ cell sort purified B cells from the spleen and bone marrow. Shown is the mean ± SEM fold increase in *Aicda* expression over C57BL/6 from three independent experiments with three mice per experiment. **(B)** The concentrations of the indicated isotypes were measured by ELISA using a purified antibody of the indicated isotype as a standard. The horizontal line represents the geometric mean antibody concentration ± 95% confidence intervals for each mouse group. Each data point represents an individual mouse.

In order to verify expression levels of *Aicda* in the *Aicda^G23S^* and 564Igi *Aicda*^G23S^ mice, we performed RT-qPCR on sorted BM and splenic B cells using AID-deficient 564Igi mice as a negative control (564Igi *Aicda*^−^*^/^*^−^). *Aicda^G23S^* mice expressed *Aicda* at levels comparable to C57BL/6 mice in both the spleen and BM, indicating that the G23S mutation in *Aicda* alone does not affect gene expression (Figure 2A). Splenic and BM B cells in 564Igi mice had elevated *Aicda* expression compared to C57BL/6 mice (Figure 2A). Similarly, 564Igi *Aicda*^G23S^ mice had significantly elevated *Aicda* levels compared to *Aicda^G23S^* mice in both the spleen and BM (Figure 2A). There was no significant difference in *Aicda* expression between 564Igi and 564Igi *Aicda*^G23S^ B cells in the spleen. However, in the BM, 564Igi *Aicda*^G23S^ mice had significantly more *Aicda* than 564Igi mice (Figure 2A).

### 564Igi *Aicda*^G23S^ Mice Have Normal IgG Antibody Titers

*Aicda^G23S^* and 564Igi *Aicda*^G23S^ mice had circulating class-switched Abs at levels equal to C57BL/6 and 564Igi mice, respectively (Figure 2B; Figure S1A in Supplementary Material). 564Igi *Aicda*^−^*^/^*^−^ mice lack all IgG isotypes (36) and were used as a negative control. 564Igi *Rag*^−^*^/^*^−^ mice lack T cells, and therefore, lack IgG1 isotype, that require T cell help (2), and thus were used as an additional control. It should be noted that C57BL/6 mice express the IgG2c allele and, therefore, secrete IgG2c Abs instead of IgG2a Abs. Because *Aicda^G23S^* mice are on the C57BL/6 background, they also express the IgG2c allele and secrete IgG2c Abs at comparable levels to C57BL/6 mice (Figure S1B in Supplementary Material). 564Igi mice, on the other hand, were generated by introducing the 564 Ig genes into embryonic stem cells derived from 129 mice, which express the IgG2a allele. Mice were then bred back onto the C57BL/6 background for more than 20 generations, selecting for the 129-derived Ig locus containing the 564 knock-in genes (2). Therefore, any mice expressing the 564Ig genes will secrete IgG2a Abs.

For the IgG2a antibody titers C57BL/6 IgH^a^ mice, which also express the IgG2a allele, were used as a control. There may be a slight decrease in serum IgG2a antibody concentrations in 564Igi *Aicda*^G23S^ mice compared to 564Igi mice, although this is not statistically significant (Figure 2B). *Ex vivo*, there is also a slight decrease in IgG2a CSR in 564Igi *Aicda*^G23S^ B cells that results in a significant decrease in IgG2a antibody secretion *ex vivo* (Figures S2A–C in Supplementary Material). While this may indicate that 564Igi *Aicda*^G23S^ B cells switch to IgG2a less efficiently than 564Igi mice, there is no difference in CSR to IgG2b or IgG1 (Figure 2B; Figures S3 and S4 in Supplementary Material). Therefore, despite increased *Aicda* expression in 564Igi *Aicda*^G23S^ mice (Figure 2A), there is no concomitant increase in general CSR activity, consistent with reports of negative regulation of *Aicda* both at the posttranscriptional and posttranslational levels (37–41).

### 564Igi *Aicda*^G23S^ Mice Have an Altered Distribution of Anti-RNA IgG Isotypes

**Figure 3:**
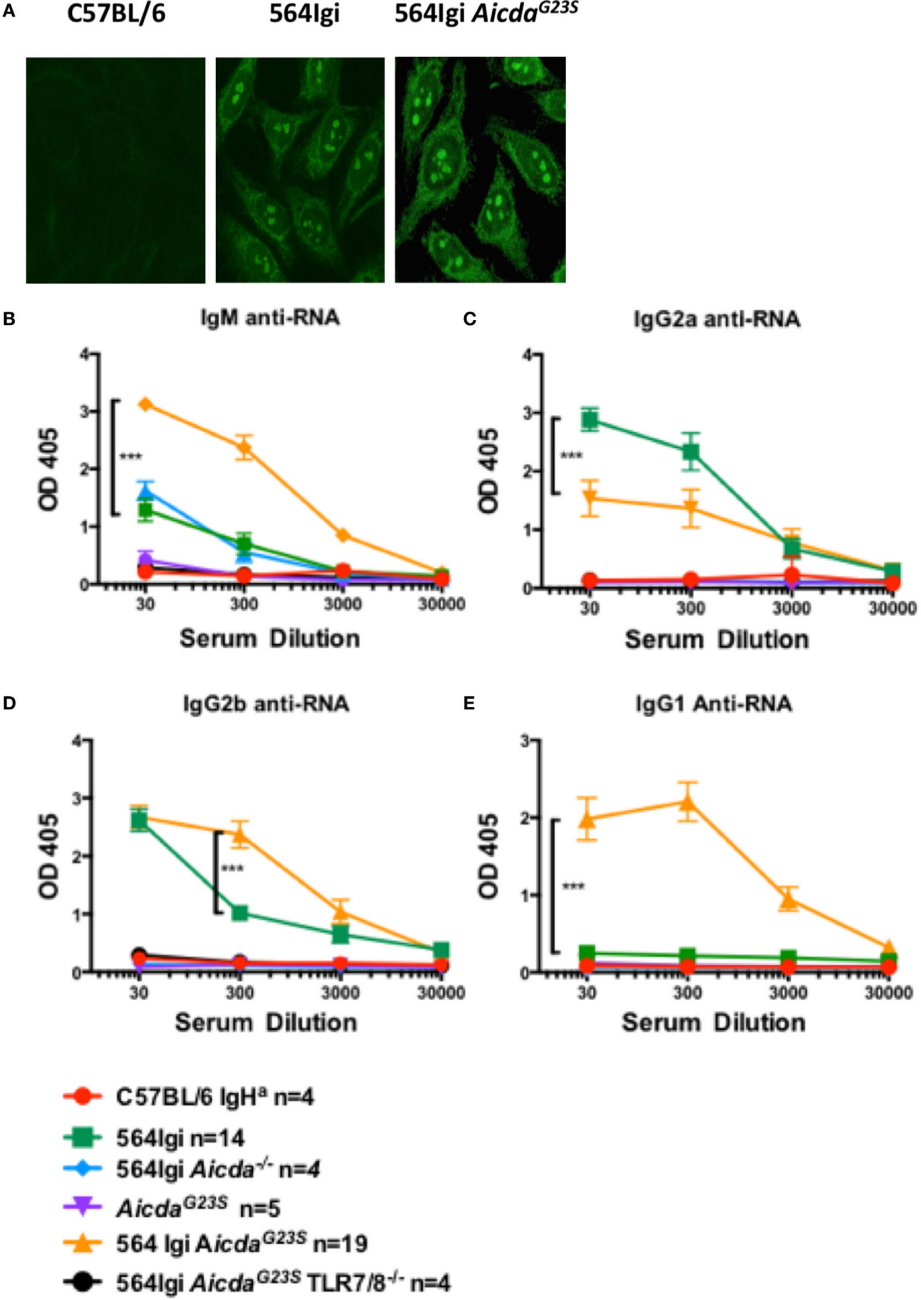
In 564Igi *Aicda^G23S^* mice most anti-RNA antibodies (Abs) are IgG2b or IgG1 while in 564Igi mice IgG2a anti-RNA Abs are predominant. **(A)** HEp-2 anti-nuclear antibody detection assays were performed with sera from the indicated mice and an anti-mouse IgG Alexa-488-labeled antibody. Shown are representative images of C57BL/6J *n* = 4, 564Igi *n* = 6, 564Igi *Aicda^G23S^ n* = 6 samples. **(B–E)** Serum anti-RNA Abs were measured by ELISA RNA binding and detection with isotype-specific for IgG2a **(B)**, IgG2b **(C)**, IgG1 **(D)**, and IgM **(E)** anti-RNA Abs. The number of mice in each group is shown in the key.

In 564Igi mice, SLE-like features have been shown to be largely mediated by IgG2a and IgG2b anti-RNA Abs that produce a characteristic nucleolar staining pattern when used for immunostaining of cells [Figure 3A; (2)]. As expected, 564Igi and 564Igi *Aicda*^G23S^ mice had elevated levels of serum anti-RNA IgG2a Abs (Figure 3B); however, 564Igi *Aicda*^G23S^ mice had less IgG2a anti-RNA Abs than 564Igi mice (Figure 3B). This decrease was not due to an overall reduction in serum IgG2a (Figure 2B). On the other hand, serum IgG2b and IgG1 anti-RNA antibody titers were elevated in 564Igi *Aicda*^G23S^ mice (Figures 3C,D). The mechanisms for these shifts in autoantibody isotype are unclear. The expression of IgG2a and IgG2b anti-RNA Abs are still dependent on TLR7/8 expression (Figures 3B,C), as has been reported for 564Igi mice (2, 3, 21). This is important as it shows that in these mice disease depends on multiple mechanisms and expression of the 564 heavy and light chain genes alone is not sufficient.

### Anti-RNA IgG2a Abs in 564Igi but Not 564Igi *Aicda*^G23S^ Mice Are Highly Mutated

**Figure 4:**
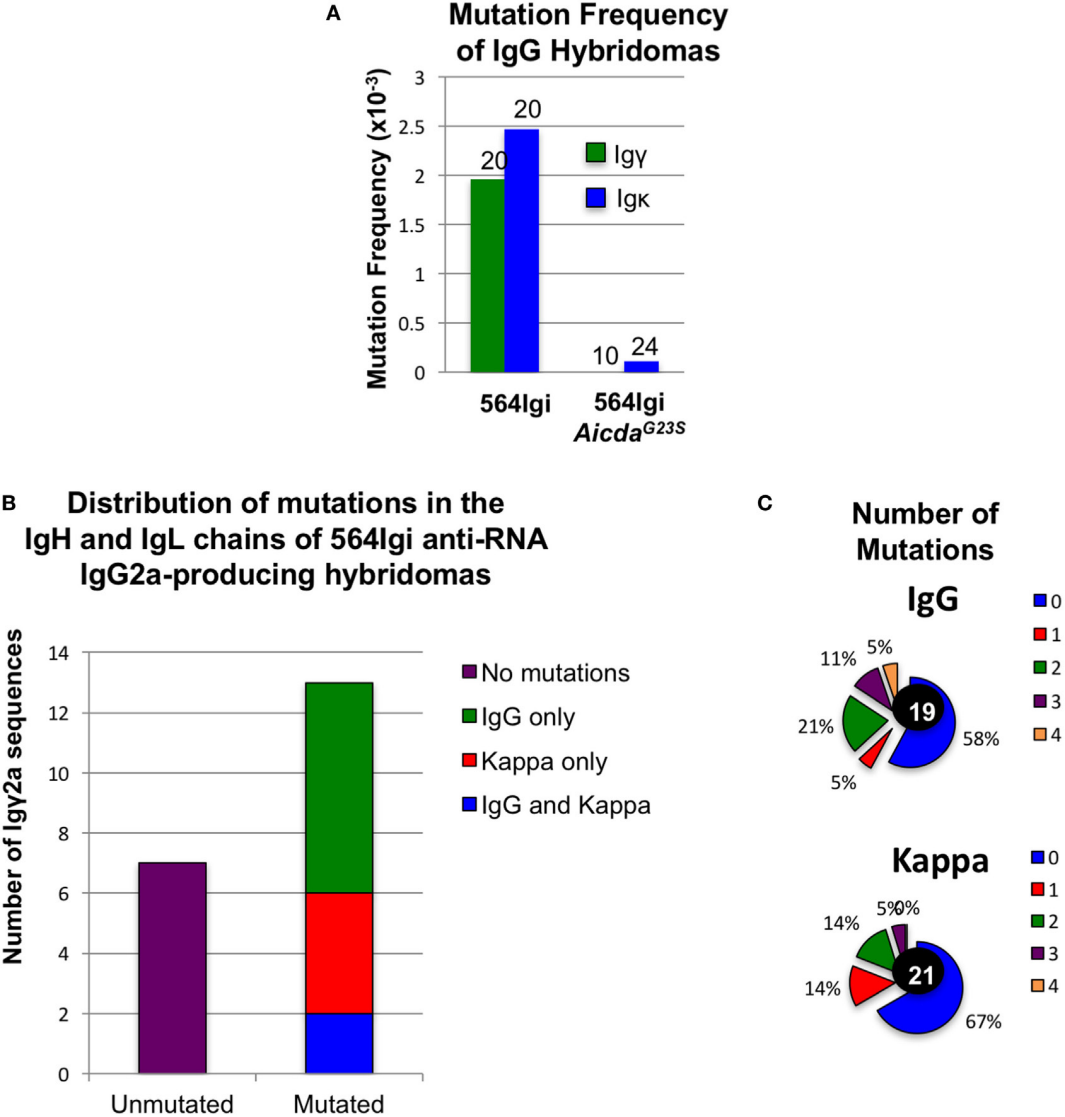
Altered isotype frequencies and reduction of somatic hypermutation in hybridomas derived from 564Igi *Aicda^G23S^* mutant mice Hybridomas were generated from the spleen and bone marrow (BM) of the indicated mice. Hybridoma culture supernatants were tested for IgG anti-RNA antibody secretion by ELISA. **(A)** The IgH and IgL genes of anti-RNA IgG-producing hybridomas from 564Igi and from 564Igi *Aicda^G23S^* mice were cloned and sequenced. Sequences were compared to the original 564 immunoglobulin knock-in sequences to identify mutations. The mutation frequency is shown. **(B)** Igγ and Igκ genes were cloned and sequenced from anti-RNA IgG2a-secreting hybridomas generated from 564Igi spleen and BM cells. Shown is the number of mutated and unmutated sequences. The number of sequences with mutations in Igγ only, Igκ only, or Igγ and Igκ is also indicated. **(C)** The percent of hybridomas from **(B)** with the indicated number of mutations in Igγ and Igκ genes is shown. The total number of sequences analyzed for each gene is shown in the center.

We sequenced Ig genes from hybridomas derived from 564Igi and 564Igi *Aicda^G23S^* mice secreting anti-RNA IgG Abs. Any hybridoma supernatant that reacted positively with RNA by ELISA was considered an anti-RNA antibody-secreting hybridoma. Only one of the Ig genes from 564Igi *Aicda^G23S^*-derived hybridomas was found to be mutated (Figure 4A). We suspect that this single mutation is most likely due to PCR error during gene amplification. These data are consistent with reports that *Aicda^G23S^* mice lack SHM (35). On the other hand, we found that 65% (13/20) of anti-RNA IgG-producing hybridomas from 564Igi mice had mutations in IgH, IgL, or both (Figures 4A–C). IgH genes generally had more mutations per gene than IgL (Figure 4C). Analyses at the single cells level from 564Igi on a RAG^−/−^ background indicated 50% of the single cells had mutations (Table 1). We suggest that the germline-encoded self reactivity of the 564Igi knock-in likely induced AID-mediated SHM during B cell development, which in turn then introduced point mutations. We more closely examined the ability of a small number of these Abs to bind RNA and found one example in which anti-RNA binding was drastically reduced compared to the original 564 antibody (Figure S5 in Supplementary Material). This study shows that SHM can result in a tolerance-inducing loss of self reactivity of the 564 antibody.

**Table 1:** Rag-mediated receptor editing in 564Igi *Aicda^G23S^* cells.

Total Igμ sequences	Mutated sequences	Unmutated sequences	VH replacements
564Igi *Aicda^G23S^*	0	26	6
33			
564Igi RAG^−/−^	25	20	0
45			
564Igi *Aicda*^−^*^/^*^−^	0	16	4
20			

*B220^+^ B cells from the indicated mice were sorted by magnetic negative selection. Igμ DNA sequences were cloned and sequenced from the bulk population and compared to the 564 immunoglobulin knock-in sequence to detect nucleotide exchanges. VH replacements were considered any sequence with more than five mutations in the VH region*.

### B Cells in 564Igi *Aicda^G23S^* GCs Actively Undergo CSR to IgG1

**Figure 5:**
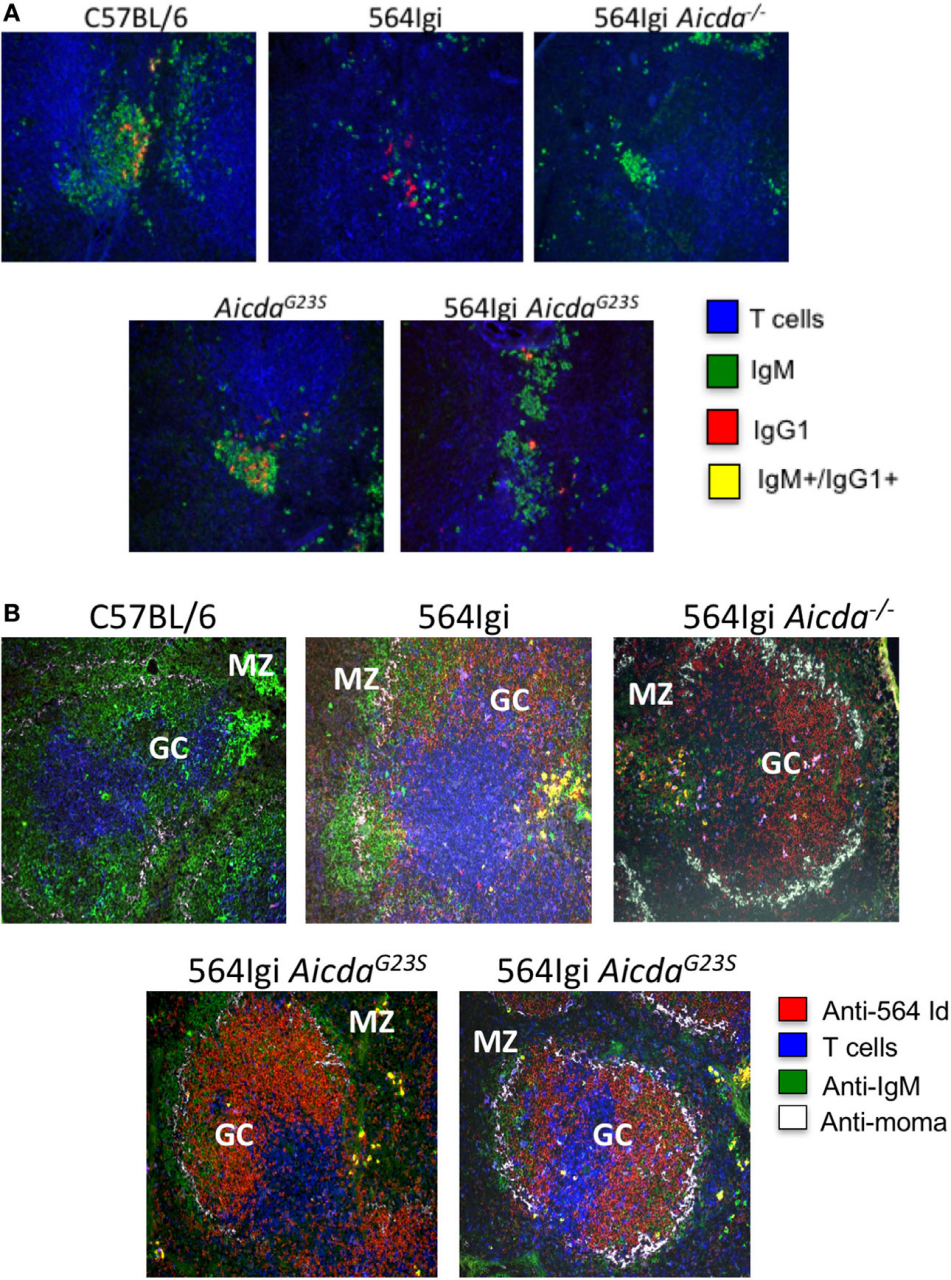
in 564Igi *Aicda^G23S^* mice germinal center (GC) B cells actively undergo CSR to IgG1. Spleen samples from the indicated mice were frozen in OCT medium, sectioned, and stained with the indicated antibodies. **(A)** Spleen sections were stained wit anti-CD4, anti-IgM, and anti-IgG1. Shown is a representative GC from each mouse. A total of three mice were analyzed for each strain. **(B)** Spleen sections were stained with anti-564 idiotype, anti-CD4, anti-IgM, and MOMA-1. The marginal zone and GC are labeled. Shown is a representative GC from each mouse. A total of two mice were analyzed for each strain.

In addition to the decrease in anti-RNA IgG2a Abs in 564Igi *Aicda^G23S^* compared to 564Igi (Figure 3B), there is a significant increase in anti-RNA IgG1 Abs (Figure 3D) of 564Igi *Aicda^G23S^*. CSR to IgG1 requires T cell help and 564Igi *Rag*^−^*^/^*^−^ mice, which lack T cells, do not secrete IgG1 Abs (Figure 2B). Therefore, anti-RNA IgG1 Abs in 564Igi *Aicda^G23S^* mice are likely to come from B cells that have received T cell help in the GC. To examine B cells in the GC, we stained for the presence of IgG1^+^ cells. 564Igi mice showed some IgG1^+^ cells, but few, if any cells that carry are both IgG^+^ and IgM^+^, which would indicate active CSR (Figure 5A). 564Igi *Aicda^G23S^* mice, on the other hand, do have IgG^+^/IgM^+^ cells in the GCs (Figure 5A), consistent with the increase in anti-RNA IgG1 Abs compared to 564Igi mice (Figure 3D). However, it has been shown that pathogenic Abs in 564Igi mice are IgG2a Abs that originate from immature B cells in the BM (2), so it is unlikely that the anti-RNA IgG1 Abs found in 564Igi *Aicda^G23S^* mice mediate pathogenesis.

### 564Igi *Aicda*^G23S^ GCs Harbor More Idiotype^+^ B Cells than 564Igi Mice

It has been shown that GCs in AID*-*deficient mice and humans are large with proliferating B cells that do not undergo apoptosis (36, 42). It has been speculated that is because in the absence of AID; the lack of CSR decreases the amount of DNA damage in the cell and thereby reduces apoptosis (42). To determine if SHM is important for GC expansion, we examined the GC phenotype of 564Igi *Aicda^G23S^* mice, that exhibit normal frequencies of CSR. We stained splenic GCs for the presence of 564 idiotype^+^ B cells. 564Igi *Aicda^G23S^* mice have many more idiotype^+^ B cells in the GC compared to 564Igi mice, similar to the phenotype of 564Igi *Aicda*^−^*^/^*^−^ mice (Figure 5B). This suggests that the lack of SHM, not the lack of CSR, may be responsible for the GC hyperplasia reported in AID-deficient mice and HIGM2 patients.

### 564Igi *Aicda*^G23S^ Females Give Birth to Few Females

We have previously showed that 564Igi females give birth to litters with high male to female ratios, a phenotype that is largely alleviated if the dams are heterozygous for the 564 IgH and IgL knock-in genes (43). Furthermore, the purified original IgG2b 564 hybridoma antibody damages developing embryos when injected into pregnant dams, resulting in litters with similarly skewed male:female ratios (43). There have been several reports showing complications during pregnancy in SLE patients (44, 45), as well as an increased incidence of cognitive disorders in the offspring born to SLE patients (46–50). In addition, the selective loss of female fetuses in animal models of SLE is due to damage by passage into the fetuses of maternal anti-nucleic acid autoantibodies that cross react with CNS *N*-methyl-d-aspartate receptors, which are more highly expressed in female fetal brains (51–53).

**Figure 6:**
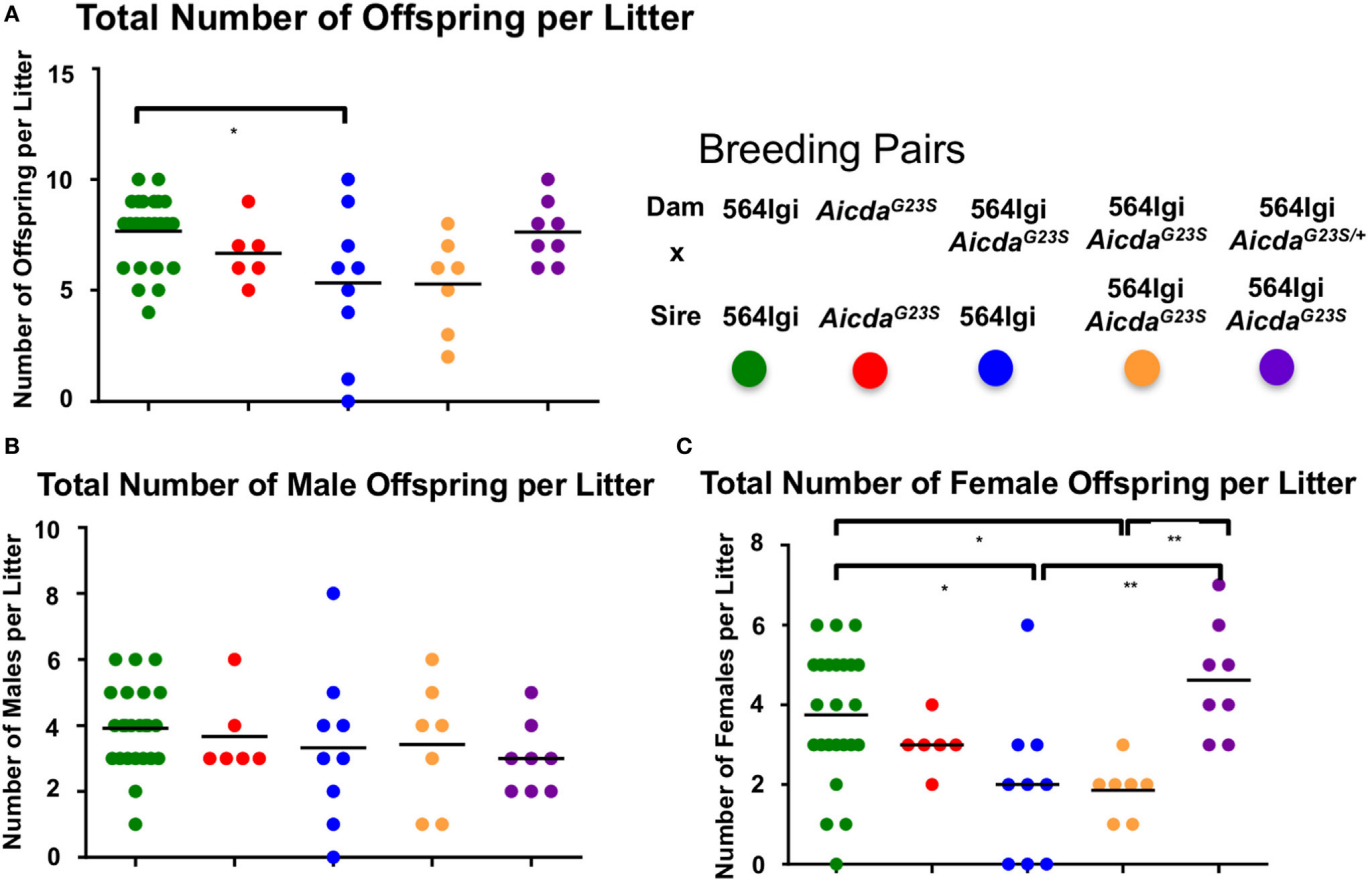
Pregnant 564Igi Aicda^G23S^ mice deliver fewer female than male offspring. The number of male and female offspring born to the indicated breeding pair were counted, and shown is the **(A)** total number of offspring, **(B)** total number of males, and **(C)** total number of females in each litter. Each data point is an individual litter born to the indicated breeding pair.

Approximately 35% of circulating anti-RNA IgG2a Abs in 564Igi mice have the original 564 H and L chain knock-in sequences (Figure 4B). We suggest that these circulating 564 Abs in 564Igi females may mediate fetal loss. 564Igi *Aicda*^G23S^ mice lack SHM and, therefore, likely have significantly higher titers of the original 564 Abs, as supported by the serum anti-RNA antibody analyses (Figure 3) and the sequence analysis of anti-RNA IgG Abs (Figure 4A). Consistent with this hypothesis, 564Igi *Aicda*^G23S^ females yield litters with elevated male:female ratios compared to 564Igi mice, regardless of the genotype of the male partner (Figure 6A, blue and orange), consistent with fetal loss due to maternal anti-nucleotide antibody. However, 564Igi *Aicda*^G23S^ females that are heterozygous for the G23S mutation (564Igi *Aicda*^G23S/+^), produce litters with male:female ratios approximately equal to 564Igi females (Figure 6A, purple). These data further point to the pathogenicity of anti-RNA Abs and suggest that SHM is a mechanism that prevents the production of autoantibodies that are pathogenic to both the adult and in the developing embryo.

### 564Igi *Aicda*^G23S^ Mice Suffer from More Severe SLE-Like Disease than 564Igi Mice

**Figure 7:**
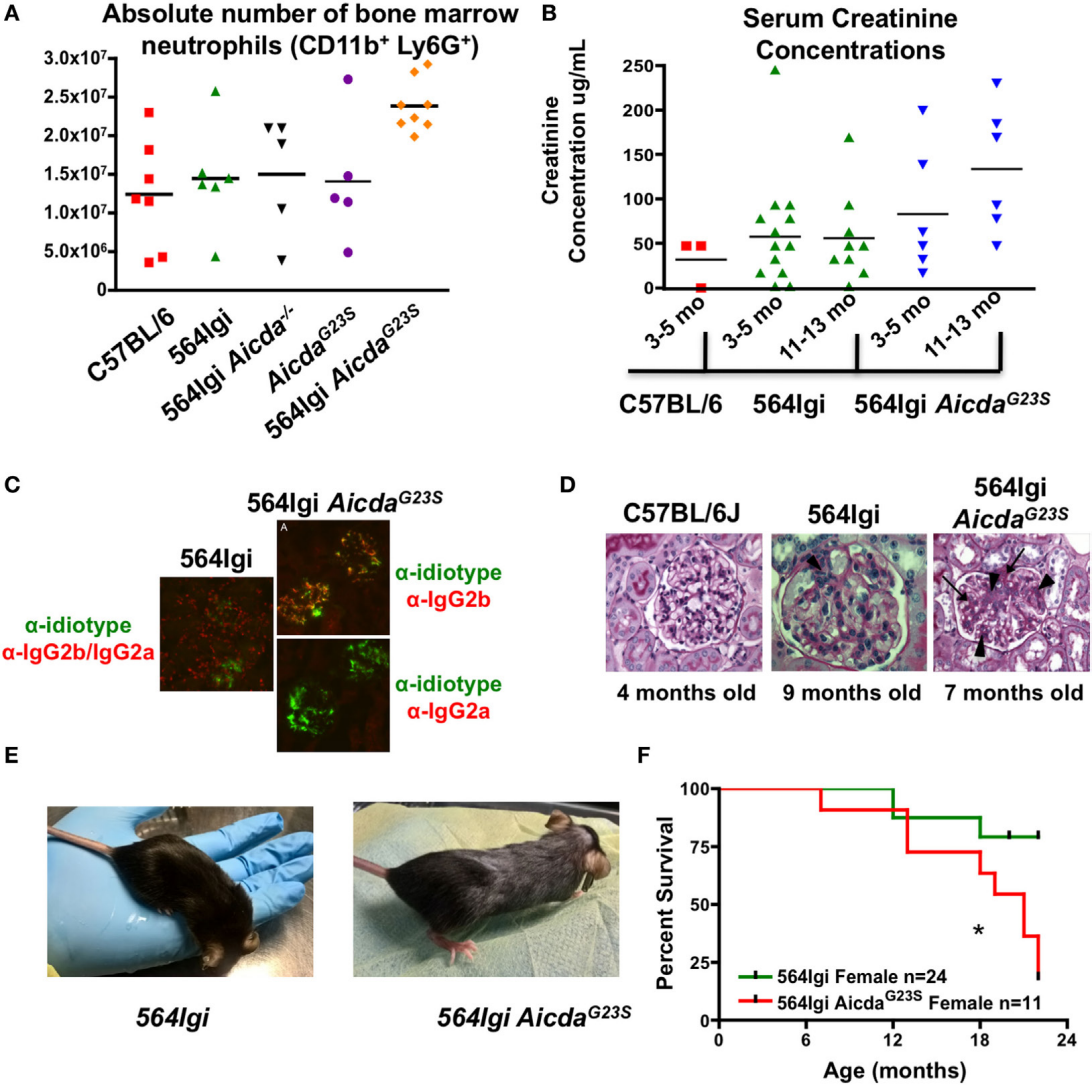
564Igi *Aicda^G23S^* mice survive poorly compared to 564Igi mice. **(A)** Bone marrow cell suspensions were stained with anti-CD11b and anti-Ly6G to detect the presence of neutrophils. The absolute number of neutrophils in from the indicated mice is shown. Each data point represents an individual animal, aged 3–6 months. The mean number of neutrophils is shown with a horizontal line. 564Igi vs 564G23S *p* < 0.02. **(B)** Serum creatinine concentrations were analyzed by ELISA. Shown is the concentration in the indicated mice at various ages. Each data point represents an individual animal. The mean concentration is shown as a horizontal line. 564Igi vs 564G23S *p* < 0.03. **(C)** Immunostaining of kidney samples from 564Igi and 564Igi *Aicda^G23S^* mice. IgG2a and IgG2b immune complexes are shown in red and 564 idiotype^+^ immune complexes are shown in green. Co-staining is seen in yellow. Shown are representative images from two mice per group. **(D)** H and E staining of kidney samples from the indicated mice. The age of each mouse is shown below the image. These are representative images of *n* = 3 mice for C57BL/6J, *n* = 5 mice for 564Igi and *n* = 5 mice for 564Igi *Aicda^G23S^*. **(E)** Skin lesions typical of 4-month-old 564Igi *Aicda^G23S^* mice. Note the loss of hair on the head and the graying of the hair on the body. **(F)** Kaplan–Meier curve showing the percent survival over time of the indicated mouse strains. The number of mice monitored for each strain is shown in the key. Statistical analysis is based on the log rank test; *p < 0.05.

Systemic lupus erythematosus pathogenesis in 564Igi mice has been shown to be mediated by neutrophil activation stimulated by antigen:antibody immunocomplexes (54). 564Igi mice have expanded neutrophil populations and an increase in IFN-I production (54). However, the increase in IFN-I is not the result of increased IFN-I secretion from individual neutrophils, but an increase in the total number of neutrophils secreting IFN-I (54). 564Igi *Aicda*^G23S^ mice have even larger neutrophil populations in the BM than 564Igi mice (Figure 7A).

As they age 564Igi mice also suffer from glomerulonephritis as they age, mediated by immune complex deposition in the renal glomeruli (2). Decreased kidney function is associated with an increase in serum creatinine concentrations. In order to test the degree of tissue damage in 564Igi *Aicda*^G23S^ mice, we measured serum creatinine concentrations as mice aged. 564Igi *Aicda*^G23S^ mice have elevated serum creatinine concentrations compared to 564Igi mice and the difference increases as mice age (Figure 7B). This suggests that 564Igi *Aicda*^G23S^ mice likely suffer from more severe kidney damage when compared to 564Igi mice.

To directly assess immune complex-mediated kidney damage, we stained kidney sections for the presence of 564 idiotype^+^ IgG immune complexes. 564Igi mice have many IgG2a/IgG2b immune complexes in the renal glomeruli, but few of these are idiotype^+^ (Figure 7C), likely due to the high level of SHM in anti-RNA IgG Abs (Figure 4). On the other hand, 564Igi *Aicda*^G23S^ mice, on the other hand, have many idiotype^+^ IgG2b but no IgG2a immune complexes in renal glomeruli (Figure 7C), consistent with serum anti-RNA antibody analyses (Figures 3B,C). Ultimately, 564Igi *Aicda*^G23S^ mice suffer from more severe glomerulonephritis than 564Igi mice (Figure 7D). The glomeruli of 564Igi *Aicda*^G23S^ mice showed an increase in mesangial cell proliferation (Figure 7D, arrow heads), matrix formation (Figure 7D, arrows/dark pink staining), and infiltration of inflammatory cells when compared to 564Igi mice. Young, 4-month-old 564Igi Aicda^G23S^ mice develop skin lesions not seen in 564Igi mice even when old (Figure 7E). 564Igi *Aicda*^G23S^ mice eventually succumb to disease more quickly than 564Igi mice (Figure 7F). Taken together, these results indicate that in the 564Igi *Aicda*^G23S^ model of SLE, SHM is not required for disease and suggest that the lack of SHM actually accelerates pathogenesis.

## Discussion

During development self-reactive B cells with surface IgM can be driven into apoptosis or anergy by ligation with self-antigen (22). However, cells that share the same developmental stage and the same specificity but have IgG rather than IgM receptors are activated by antigenic ligation and, therefore, escape tolerance (24, 25). Therefore, because the consequences of ligation of antibody and antigen vary with the isotype of the antibody, AID, by enabling CSR, can enable a self-reactive B cell to escape deletion.

Previously, we showed that production of IgG autoantibody in the 564Igi model of SLE requires expression of activation-induced deaminase in early-developing B cells (3, 21). This is not likely to be unique to 564Igi mice as AID is expressed in normal developing B cells in several strains (12). Other reports indicate that SHM can generate autoantibodies (55–57), but it is not yet known if this SHM contributes to the generation of pathogenic autoantibody. Nevertheless, AID through CSR or SHM, can generate autoantibody. Further, prior attempts to demonstrate directly that AID-dependent SHM is crucial for the development of pathogenic autoantibody have been hampered by the utilization of knockout mice in which both SHM and CSR are lost (58). Thus, it is no doubt true that SHM can generate high affinity anti-DNA autoantibodies, but care must be taken to not confuse “high affinity” autoantibody with “pathogenic” autoantibody. By the latter, we mean antibody that has been shown to induce pathology when transferred to otherwise normal mice. Also, because the DNA-specific TLR9 acts as a negative regulator of autoantibody production (59–62), it is unlikely that DNA as antigen drives autoimmunity. In fact in the absence of TLR9, MRL/lpr mice make no anti-DNA antibodies, yet there is no mitigation of their severe glomerulonephritis; suggesting that mouse lupus does not require anti-DNA antibodies (59).

Several groups have shown that AID can contribute to the loss of self reactivity in mice and humans (16, 33, 34, 63, 64, 65). Furthermore, a recent interesting report by Goodnow’s group showed that in humans *in vivo* SHM of germ line genes encoding an autoantibody results in loss of self reactivity (66). These studies did not address whether AID-dependent tolerance actually provides protection against pathogenic antibody. Nor did they show directly that AID-dependent SHM was required. The studies reported here address these issues directly and AID-dependent SHM is crucial for a B cell tolerance mechanism to limit the secretion of autoantibodies and to prevent the progression of SLE. Yet, at the same time, AID-dependent CSR is required for the production of these pathogenic IgG autoantibodies.

Given this ability to affect the production of autoantibodies, multiple studies have used knockout strategies to address the role of AID in systemic autoimmunity. In MRL/lpr mice, however, the absence of AID results in the production of protective IgM Abs and amelioration of autoimmune disease (67). Thus, the relative contribution of SHM and CSR to the generation of autoantibodies in SLE-like disease is unclear.

In the BXD2 model of autoimmunity a dominant negative mutant of *Aicda* was introduced as a transgene, mitigating the disease (68). However, although the intent was to block only the SHM functions of AID, the mutation compromised class switching and, as a result, the study did not address the original question. In *Aicda*^−/−^ BALB/c mice, organ-specific but not systemic autoimmunity develops in older animals (69). In MRL/lpr mice with deletion of *Aicda* there was a significant increase in autoreactive IgM Abs against various antigens, a decrease in IgG Abs to these antigens and a mitigation of disease. Passive transfer of IgM Abs to dsDNA from these mice to otherwise unmanipulated MRL/lpr mice provided protection (70).

Studies in humans agree with the animal model work as they do not provide evidence that the absence of *Aicda* is sufficient to cause systemic autoimmune disease. While AID-deficient humans with hyper-IgM have an increased risk of autoimmune and inflammatory disorders such as diabetes mellitus, polyarthritis, autoimmune hepatitis, hemolytic anemia, immune thrombocytopenia, Crohn’s disease, and chronic uveitis, there is no clear documentation of SLE in these patients (71).

Manifestations of SLE, including glomerulonephritis, are mainly mediated by IgG2a anti-RNA Abs that are produced by B cells that develop in the BM of 564Igi mice (2). Here, we show that in 564Igi *Aicda*^G23S^ mice disease is also mediated by IgG2a/IgG2b Abs. These Ig subclasses bind the activating Fc receptors, FcγRIV (IgG2a and IgG2b); FcγRI (IgG2a only) with high affinity (72). IgG1, on the other hand, binds only the Fc receptor FcγRIII (73) with low affinity. Thus, all activating mouse FcγRs (FcγRI, FcγRIII, and FcγRIV) bind to IgG2a and IgG2b, whereas FcγRIII is the only activating FcγR that binds IgG1 and FcγRI is the only activating FcγR that binds IgG3. The production of IgG1 requires T cell help and is, therefore, only produced by mature B cells in the GC. Therefore, the most pathogenic Abs in 564Igi *Aicda*^G23S^ mice are likely to be IgG2a/IgG2b Abs produced by developing B cells. Because IgG1 does not bind activating FcR we believe that it does not contribute to the pathology.

FcγRIIb, the inhibitory Fc receptor, induces pro-apoptotic signals in mature B cells, acting as a peripheral tolerance mechanism. However, co-ligation of FcγRIIb and the BCR by a high affinity antigen–antibody complex may bypass apoptotic signals and initiate positive selection of those cells in the GC (74). In the BM of 564Igi and 564Igi *Aicda*^G23S^ mice, the presence of self-reactive Abs likely induces *Aicda* expression, as described above. In 564Igi mice, AID-mediated SHM may decrease the affinity of the BCR for antigen. In combination with FcγRIIb engagement by IgG2a Abs produced by BM B cells, many self-reactive cells likely undergo apoptosis. However, in 564Igi *Aicda*^G23S^ mice, the lack of SHM causes a higher affinity of the BCR for antigen, which in combination with FcγRIIb engagement induces a stimulatory signal to further increase autoantibody production.

Previous reports have shown that the *Aicda^G23S^* mutation results in increased bacterial load in the small intestine of naïve mice (35). In addition, the *Aicda^G23S^* mutation decreases survival of mice orally challenged with cholera toxin and increases the bacterial load of mesenteric lymph nodes of mice fed *Y. enterocolitica*, correlating SHM with the capacity to protect the mucosa (35). To avoid the impact of an altered microbiome on our experiments, the mice were placed on oral Uniprim, which contains sulfadiazine and trimethoprim. However, the effect of microbial load on the survival of naïve *Aicda^G23S^* mice has not been examined. We did not see evidence of disease in *Aicda*^G23S^ mice that did not carry 564 H and L chain gene knock-ins.

It has been shown that antibody constant regions can also impact antibody pathogenicity (75). This suggests that the fine affinities of the IgG2a/IgG2b/IgG1 anti-RNA Abs may vary slightly due to differences in the constant regions and affect the pathogenesis of SLE in 564Igi and 564Igi *Aicda*^G23S^ mice. The receptors in 564Igi *Aicda*^G23S^ mice may have a higher affinity for RNA *in vivo*, which causes the B cell to become activated, to migrate to the GC and to undergo CSR to IgG1 with T cell help. In 564Igi mice, on the other hand, the introduction of nucleotide exchanges in anti-RNA receptors may alter antibody affinity and/or specificity for antigen *in vivo*, promoting the selection of IgG2a^+^ cells in a T cell-independent manner. These mutated Abs may be less pathogenic *in vivo* compared to the original Abs in 564Igi *Aicda*^G23S^ mice, allowing 564Igi mice to survive longer. This hypothesis is supported by the fact that circulating maternal Abs in 564Igi females seem to be less pathogenic than those in 564Igi *Aicda*^G23S^ females (Figure 6).

In sum, in the 564Igi mice, the loss of SHM results in more severe autoimmunity and early death, suggesting that AID can mitigate autoantibody production by altering antibody specificity.

## Experimental Procedures

### Mice

All experiments with mice were performed in accordance with the regulations of and with the approval off the Tufts/TMC IACUC (protocol B2015-41). Creation of the 564Igi mice was previously described (2). 564Igi mice have knocked-in H and L chain genes of the 564 hybridoma derived from an (SWRxNZB)F_1_, mouse, a known model of SLE. The H chain of 564 has three nucleotide replacements, none in the CDRs, compared to the germ line of SWR (76). The 564 L chain gene has two nucleotide replacements, in the CDR2 when compared to 45–21.1 (76, 77). All 564Igi mice are homozygous for the 564 IgH and IgL alleles unless otherwise indicated. *Aicda*^−^*^/^*^−^ and *Aicda^G23S^* mice were obtained from Dr. T. Honjo (Kyoto University, Japan). C57BL/6, C57BL/6 IgH^a^, and *Rag2*^−^*^/^*^−^ mice were purchased from Jackson Laboratories. All mice were genotyped by Transnetyx Inc. Experiments were performed with male and female mice unless otherwise indicated. All mice in this study were maintained on a diet that included Uniprim^®^, a combination of sulfadiazine and trimethoprim, to prevent an expansion of microflora in the small intestine as seen in untreated mice expressing the G23S mutant form of AID (35).

### qRT-PCR

RNA was isolated from cells by TRIzol incubation (Life Technologies 15596) followed by chloroform extraction. All RT-qPCR experiments were performed using two fourfold serial dilutions of RNA and iScript Reverse Transcription Supermix for RT-qPCR from Bio-Rad (170-8841). Triplicate cDNA samples were used for the amplification of *Actb* and *Aicda* using commercially available FAM primer probes (Life Technologies) and a Bio-Rad IQ5 quantitative PCR system. Gene expression was normalized to *Actb* expression based on a standard curve.

### Cloning and Sequencing

Sequencing was performed as previously described (21). Briefly, single CD138^+^ cell or a population of B220^+^ cells were sorted by flow cytometry. RNA was isolated and converted into cDNA using the SuperScript III First Strand Synthesis System (ThermoFisher 18080051) or TRIZOL reagent followed by the iScript cDNA Synthesis Kit (BioRad 170-8890), for single cells and cell populations, respectively. Ig genes were then amplified, cloned, and expressed as described (21).

### ELISAs

#### Total Isotype/Anti-RNA

ELISAs were performed as previously described (21).

#### Serum Creatinine

Serum creatinine concentrations were determined using a commercially available kit from Abcam (ab65340) following the manufacturer’s protocol.

### Hybridomas

Spleen- or BM-derived mouse cells were fused with the P3X63Ag8.653 mouse myeloma cell line (78). Cells were grown in 96-well culture plates in media to select for the growth of successfully fused cells (15% FCS, 10% hypoxanthine and 10% azaserine RPMI complete media). After 2 weeks, culture supernatants from healthy hybridomas were collected and tested for antibody production by ELISA and cells were collected for cloning and sequencing.

### Flow Cytometry

Cells were stained for flow cytometry according to standard procedures. Single cell suspensions were diluted to 1 × 10^6^ cells/mL in FACS staining buffer (1% heat-inactivated rabbit serum/0.1% NaN_3_/1× DPBS with Ca^++^ Mg^++^). Cells were centrifuged and resuspended in 50 µL of fluorescent Abs (Southern Biotech and Biolegend) diluted to 1 µg/mL in FACS staining buffer. Samples were washed in 2 mL FACS staining buffer and resuspended in 500 µL FACS staining buffer for analysis. Propidium iodide was added to a final concentration of 10 ng/mL just prior to analysis on a FACScalibur flow cytometer (BD Biosciences) to assess cell viability.

### HEp-2

Anti-nuclear Abs were detected by HEp-2 staining according to the manufacturer’s protocol (MBL Bion ANK-120).

### Immunofluorescence and Light Microscopy

Fresh kidney or spleen samples for immunofluorescence studies were frozen in OCT medium. 4 µm sections were cut on a cryostat and mounted on a glass slide. The sections were air dried for 1 h, rehydrated in PBS, and incubated with anti-IgG2a/IgG2b/IgG1/IgM, anti-CD4, anti-MOMA, and anti-idiotype Abs for 1 h at room temperature. The sections were rinsed again in PBS, mounted in Fluoromount (Southern Biotech), and examined and photographed with a Leica fluorescence microscope. For light microscopy studies, fresh kidneys were fixed in 10% buffered formalin and embedded in paraffin. 5 µm paraffin sections were stained with periodic acid-Schiff and evaluated by light microscopy.

### Statistical Analysis

The *p* values were calculated using one-way ANOVA for all analyses, followed by the Tukey multiple comparison test unless otherwise indicated (Prism GraphPad Software). For all figures *p < 0.05, **p < 0.01, and ***p < 0.001.

## Ethics Statement

All experiments with mice were performed in accordance with the regulations of and with the approval of the Tufts/TMC IACUC (protocol B2012-41).

## Author Contributions

GM designed the study with TI-K and carried out experiments, some of them with CM, did statistical evaluations, analyzed the data, and wrote the manuscript; MP was responsible for histology and analysis of pathology in Kidneys and spleens. CM with GM performed experiments with single cells, cloned and sequenced H and L chain genes, and expressed functional H and l chain genes. Analyzed data with GM and TI-K. JK did immunocytochemistry and fluorescent analysis of spleen and kidney sections of mice. RS was responsible for the generation of Aicda-mutant mice. TH was responsible for the idea and creation of Aicda mutant mice unable to SHM Ig genes, but capable of CSR. ES together with TI-K and GM was involved in the design and critical analysis of the data. GM and TI-K wrote the manuscript, and ES and HW critically reviewed the data and added comments and revised the manuscript.

## Conflict of Interest Statement

The authors declare that the research was conducted in the absence of any commercial or financial relationships that could be construed as a potential conflict of interest.
