# Supplementary figures and images for: Accelerated Systemic Autoimmunity in the Absence of Somatic Hypermutation in 564Igi: A Mouse Model of Systemic Lupus with Knocked-In Heavy and Light Chain Genes
Source: Front Immunol. 2017 Sep 13;8:1094. doi: 10.3389/fimmu.2017.01094 (PMC5601273; doi:10.3389/fimmu.2017.01094)

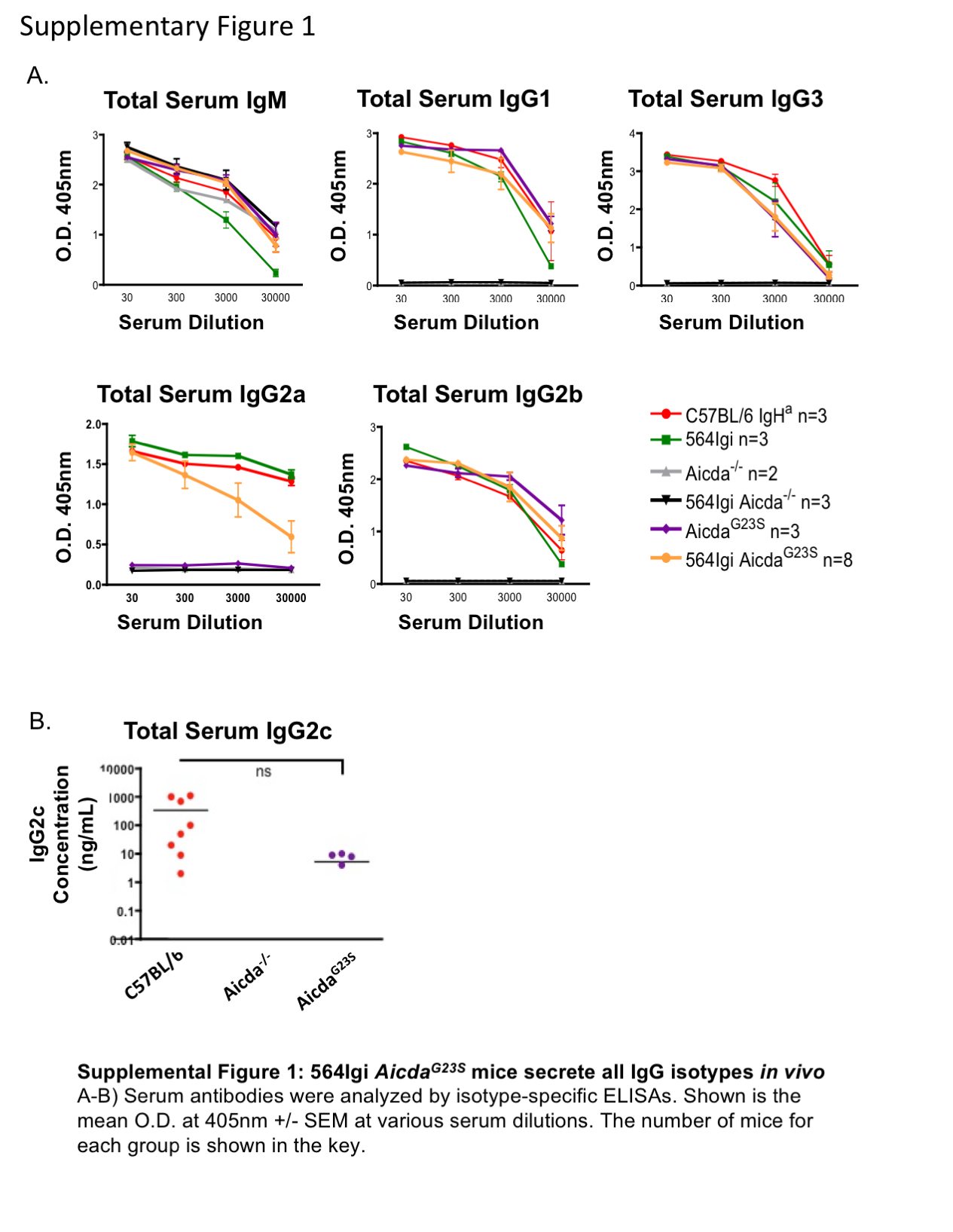

Supplement: Supplementary file 1 [file image_1.tiff]

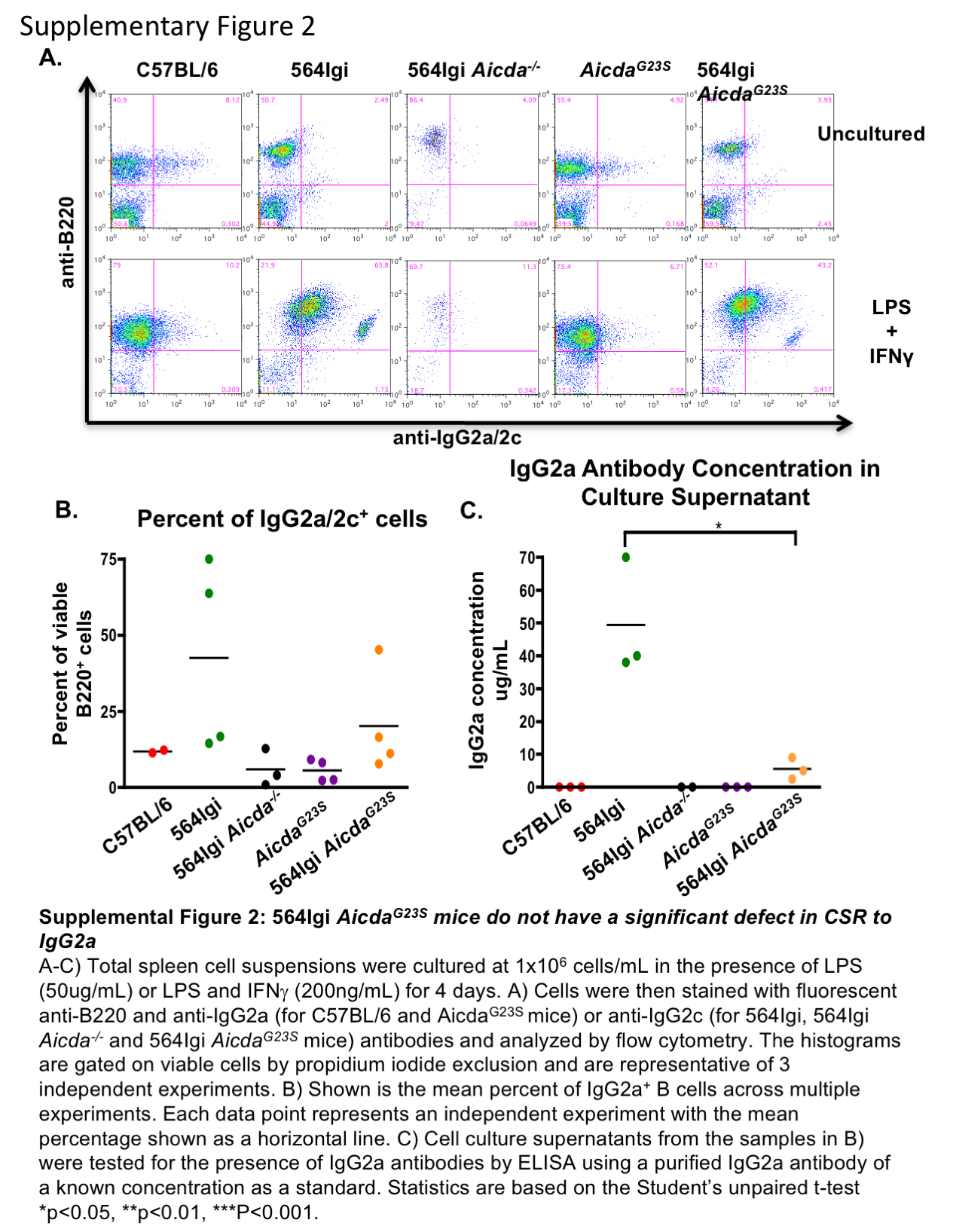

Supplement: Supplementary file 2 [file image_2.tiff]

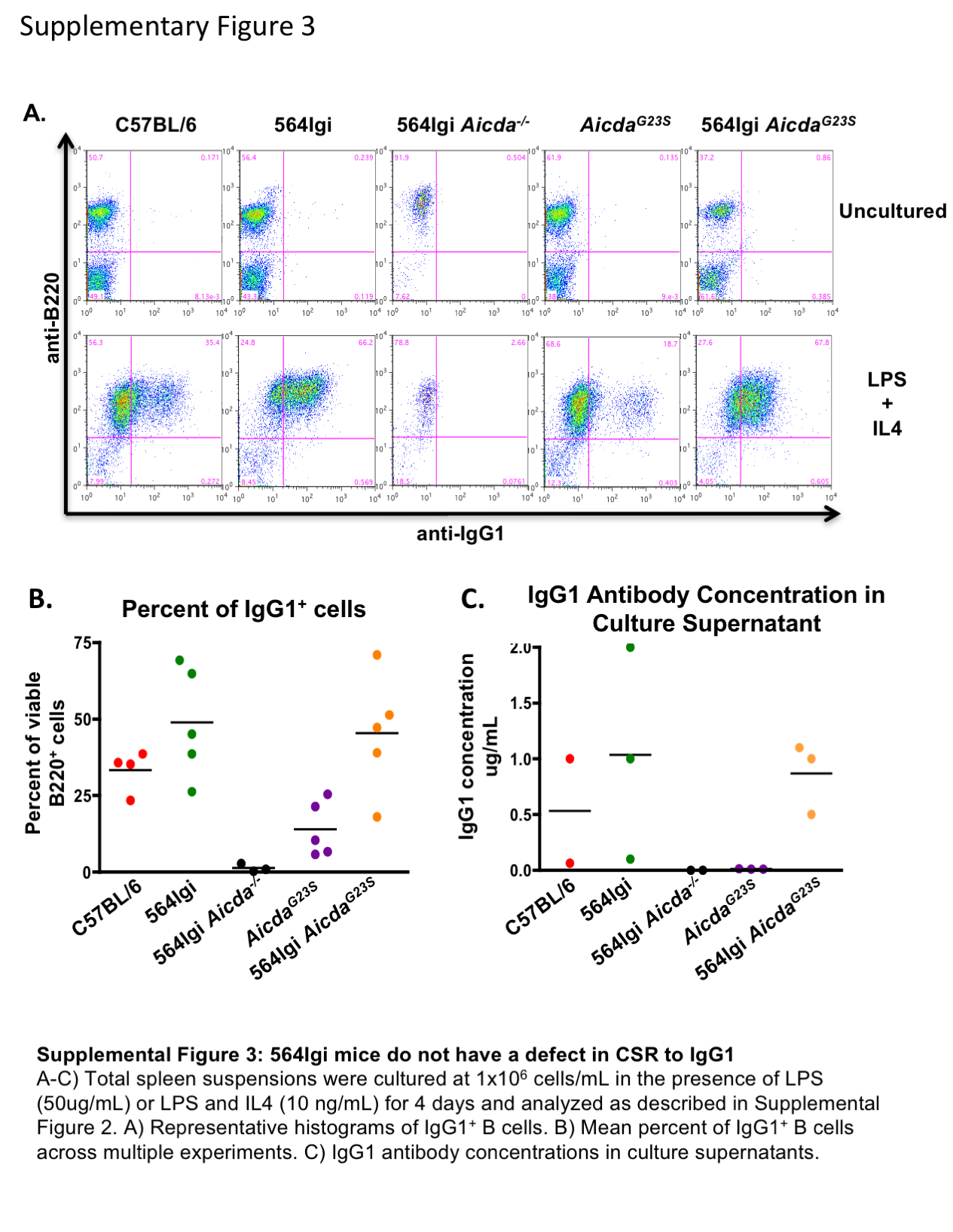

Supplement: Supplementary file 3 [file image_3.tiff]

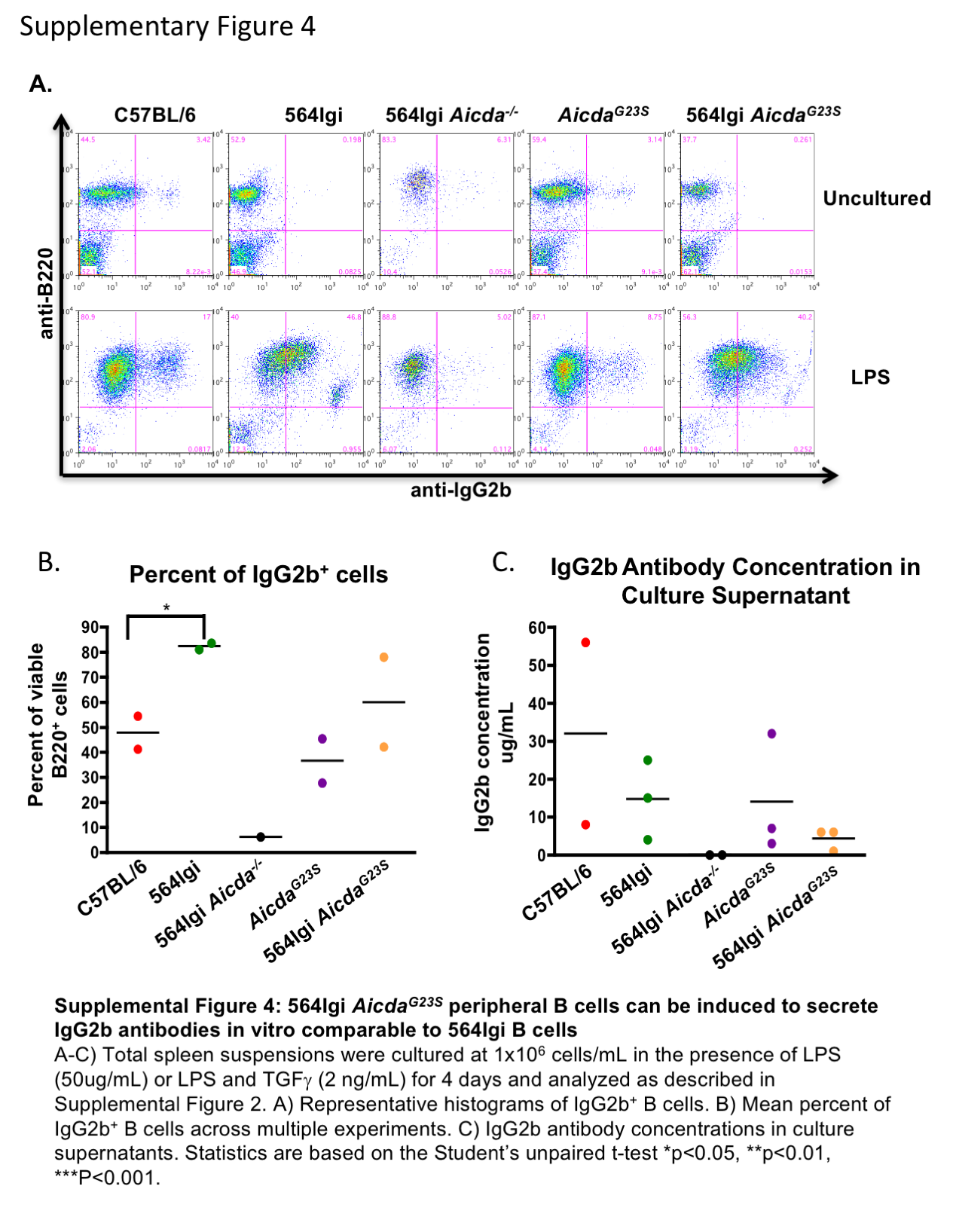

Supplement: Supplementary file 4 [file image_4.tiff]

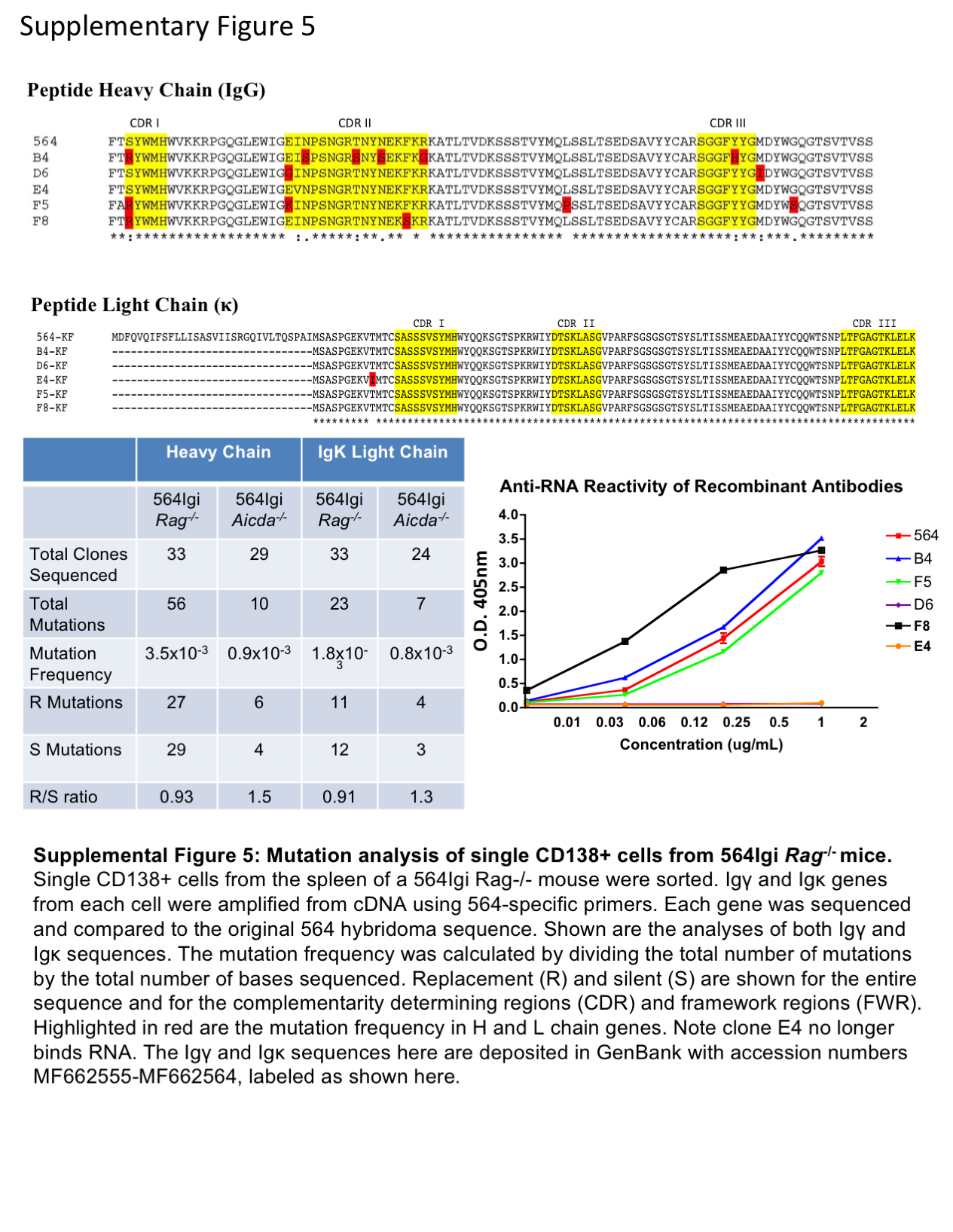

Supplement: Supplementary file 5 [file image_5.tiff]
